# Optimized algorithm for speed‐of‐sound‐based infant sulfur hexafluoride multiple‐breath washout measurements

**DOI:** 10.1002/ppul.27180

**Published:** 2024-07-18

**Authors:** Florian Wyler, Thuvarakha Manogaran, Nathalie Monney, Yasmin Salem, Ruth Steinberg, Anne‐Christianne Kentgens, Carvern Jacobs, Shaakira Chaya, Carla Rebeca da Silva Sena, Noëmi Künstle, Olga Gorlanova, Sophie Yammine, Diane M. Gray, Urs Frey, Marc‐Alexander Oestreich, Philipp Latzin

**Affiliations:** ^1^ Division of Paediatric Respiratory Medicine and Allergology, Department of Paediatrics, Inselspital, Bern University Hospital University of Bern Bern Switzerland; ^2^ Graduate School for Cellular and Biomedical Sciences University of Bern Bern Switzerland; ^3^ Graduate School for Health Sciences University of Bern Bern Switzerland; ^4^ Department of Paediatrics and Child Health Red Cross War Memorial Children's Hospital Cape Town South Africa; ^5^ University Children's Hospital Basel UKBB University of Basel Basel Switzerland

**Keywords:** lung clearance index, OASIS, WBreath

## Abstract

**Introduction:**

Major methodological issues with the existing algorithm (WBreath) used for the analysis of speed‐of‐sound‐based infant sulfur hexafluoride (SF_6_) multiple‐breath washout (MBW) measurements lead to implausible results and complicate the comparison between different age groups and centers.

**Methods:**

We developed OASIS—a novel algorithm to analyze speed‐of‐sound‐based infant SF_6_ MBW measurements. This algorithm uses known context of the measurements to replace the dependence of WBreath on model input parameters. We validated the functional residual capacity (FRC) measurement accuracy of this new algorithm in vitro, and investigated its use in existing infant MBW data sets from different infant cohorts from Switzerland and South Africa.

**Results:**

In vitro, OASIS managed to outperform WBreath at FRC measurement accuracy, lowering mean (SD) absolute error from 5.1 (3.2) % to 2.1 (1.6) % across volumes relevant for the infant age range, in variable temperature, respiratory rate, tidal volume and ventilation inhomogeneity conditions. We showed that changes in the input parameters to WBreath had a major impact on MBW results, a methodological drawback which does not exist in the new algorithm. OASIS produced more plausible results than WBreath in longitudinal tracking of lung clearance index (LCI), provided improved measurement stability in LCI over time, and improved comparability between centers.

**Discussion:**

This new algorithm represents a meaningful advance in obtaining results from a legacy system of lung function measurement by allowing a single method to analyze measurements from different age groups and centers.

## 1 INTRODUCTION

Multiple‐breath washout (MBW) is a method to measure ventilation inhomogeneity in the lungs.^1^ The test measures the volume of breathing required to replace the gas mixture present in the lungs at the beginning of the test with a new mixture (cumulative expired volume [CEV]). The test ends when the concentration of the tracer gas reaches 2.5% of the initial concentration. The CEV, when normalized with the functional residual capacity (FRC), yields the test's primary outcome: the lung clearance index (LCI).^1^ In older children and adults, nitrogen (N_2_)‐MBW is an increasingly used and established method,^2^, ^3^ and the LCI has been shown to be a sensitive marker of early structural lung disease in children with cystic fibrosis (CF).^4^

Infant MBW, typically performed by washing a mixture of air and sulfur hexafluoride (SF_6_) in and out of the lungs, is feasible in natural sleep and under sedation, but its use has been limited by technical and methodological issues.^5^, ^6^, ^7^ The first commercially available setup for infant MBW was a combination of an ultrasonic flow meter ^8^ to measure main‐stream molar mass signals (Exhalyzer D, Ecomedics AG), which were analyzed using software called WBreath (ndd Medizintechnik AG).^9^ Measurements using this technology have been carried out in cohort studies in centers across the globe.^10^, ^11^, ^12^

There are major methodological issues with the algorithm which WBreath uses for the analysis of molar mass‐based infant SF_6_ MBW measurements. Outcomes are highly dependent on a large set of input parameters (e.g., room and body temperature, humidity, time, and diffusion constants) which depend partly on the environment and the test subject, and therefore need to be adjusted between centers and different groups being measured.^7^, ^13^, ^14^ This means that the analysis of data collected in a different environment or measuring a new subject group requires renewed validation for each set of input parameters.

Small deviations in input parameters can greatly affect results, or even make it impossible to obtain results from measurements.^15^ These issues make the outcomes of the measurements unreliable, lead to implausible results, and complicate the comparison between different age groups or centers.^16^

To address this, we aimed to develop a new algorithm to analyze molar mass signals originating from infant SF_6_ MBW measurements, and validate this new algorithm in three ways:

1. For in vitro FRC measurement accuracy under variable temperature (T), respiratory rate (RR), tidal volume (VT), ventilation inhomogeneity (VI), and target FRC conditions,
2. In vivo by demonstrating feasibility as well as independence from difficult‐to‐validate temperature and diffusion settings on analysis outcomes,
3. In vivo by demonstrating that the new algorithm produces more plausible results in existing data sets where the use of WBreath leads to questionable results (step changes in longitudinal tracking of LCI and within measurement series over time, lack of comparability between centers).

## 2 METHODS

### 2.1 In vitro measurements

To validate functional residual capacity (FRC) measurement accuracy in vitro, we collected measurements in an infant Plexiglas lung model described previously.^17^, ^18^ In two FRC target volumes (80 mL, 210 mL), we varied the temperature of the surrounding water bath, the respiratory rate, tidal volume, and ventilation inhomogeneity via a perforated metal grate insert creating two subdivisions in the lung model with differential ventilation (Supporting Information: Table S1). We chose FRC target volumes to be representative of the first year of life. Assuming FRC values of 22 mL/kg, this corresponds to infants in the weight range of 3.6–9.5 kg.^7^ Each of the combinations of FRC volumes and conditions was measured six times (one original triplicate, and a repeat triplicate on a new calibration).

### 2.2 Retrospective in vivo data

For the investigation of the impact of the new algorithm on in vivo data, we used retrospective data from the Basel‐Bern Infant Lung Development (BILD), the Swiss Cystic Fibrosis Infant Lung Development (SCILD), and Drakenstein Child Health cohorts, described elsewhere.^10^, ^11^, ^19^, ^20^ These data include:

1. Healthy infants measured at around 6 weeks (Bern, *n* = 62), and children with CF measured at around 8 weeks, 1 year, and as long‐term validation in a follow‐up at 6 years (Bern, *n* = 21).
2. Healthy infants measured at around 6 weeks (Basel, *n* = 78).
3. Healthy infants measured at around 8 weeks and 1 year (Drakenstein, *n* = 48).

A detailed overview of the data sets is provided in Table 1. Parents gave informed written consent, and the respective study protocols were approved by the ethics committees of the Canton of Bern (2019‐01072 and 2017‐02139), Northwest and Central Switzerland (2022–00336) as well as the Western Cape Provincial Health Research Committee and University of Cape Town Human Research Ethics Committee (401/2009).

**Table 1:** Demographics for the retrospective in vivo data sets.

Center	SA	SA	Bern	Bern	Bern	Basel
Age group	Infant	Toddler	Infant	Infant	Toddler	Infant
HC/CF	HC	HC	HC	CF	CF	HC
*n*	48	48	62	21	21	78
Age birth [w]	39.6 (1.7)	39.6 (1.7)	39.7 (1.1)	38.9 (1.6)	38.9 (1.6)	39.8 (1.0)
Age test [d]	54 (8.0)	383 (24)	40 (9.5)	59 (15)	387 (22)	34 (5.1)
Length [cm]	56 (2.5)	74 (2.9)	56 (4.5)	55 (2.3)	74 (2.1)	55 (2.7)
Weight [kg]	5.0 (0.7)	9.6 (1.3)	4.5 (0.6)	4.5 (0.7)	9.1 (1.0)	4.4 (0.6)

*Note*: Numbers are provided as mean (SD) of study population. Age birth: Gestational age at birth in weeks. Age test: Postnatal age at test in days.

Abbreviations: CF, cystic fibrosis; d, days; HC, healthy control; SA, South Africa; SD, standard deviation; w, weeks.

### 2.3 MBW analysis

Infant MBW measurements were performed as described previously.^7^, ^20^ We used two configurations for the analysis of raw MBW data in WBreath analysis software, reflecting the current standards of the Drakenstein (config I) and BILD/SCILD (config II) cohorts, respectively (Supporting Information: Table S2).^7^, ^20^ When using the new algorithm, total dead space was set for 4.6 and 9.6 ml for infant and toddler age groups respectively (Table 1).

Study visits were considered acceptable if there were at least two measurements with acceptable visual quality control which produced LCI and FRC outcomes within 25% of the measurement mean.

Long‐term follow‐up of infants with CF at age 6 years was measured with N_2_MBW using the Exhalyzer D (Ecomedics, Duernten, Switzerland), and analyzed using Spiroware 3.3.1.

### 2.4 OASIS algorithm description

The new algorithm OASIS (Optimized Algorithm for Speed‐of‐sound‐based Infant Sulfur hexafluoride multiple‐breath‐washout) deals with extracting an SF_6_ signal from speed of sound‐based molar mass signals collected using an ultrasonic flow meter in infant MBW measurements (Supporting Information: Figure S1). The algorithm replicates the functionality of WBreath, while minimizing the algorithm's dependence on temperature and diffusion models.

The algorithm consists of the following steps:

1. Breath and phase detection.^21^
2. Tidal change correction (replacing temperature simulation).
3. Side chamber correction (replacing step response).
4. BTPS correction of flow signal.
5. Outcome calculation.

The entire algorithm is available as a MATLAB 2021a script (MathWorks) in the online supplement, and steps (i) and (iv) do not conceptually differ from the equivalent steps in the WBreath analysis software. Step (v) is simplified in OASIS, as it directly produces an SF_6_ signal, rather than a corrected molar mass signal as WBreath does.^7^, ^15^ The tidal change and side chamber corrections attempt to solve the two major issues with the raw data collection using the ultrasonic flow meter:

1. Temperature, humidity, and changes in oxygen (O_2_)/carbon dioxide (CO_2_) concentration cause confounding contributions to the raw molar mass signal that must be accounted for to accurately measure SF_6_.
2. Gas trapped in the side chambers contributes to the raw measured molar mass signal, and must be isolated to accurately measure only the gases being washed in or out.

### 2.5 OASIS: Tidal change correction

The ultrasonic flow meter measures molar mass via the speed of sound of the medium in its measurement path (Supporting Information: Figure S1). The speed of sound and molar mass are related according to:

(1) $$c=\sqrt{\frac{\gamma \;*\;R\;*\;T}{\mathrm{MM}}}.$$

Equation 1: The relationship between speed of sound, temperature, and molar mass. *c*: speed of sound, *γ*: adiabatic index, *R*: gas constant, *T*: temperature, and *MM*: molar mass.

The molar mass signal is therefore dependent not only on the washin or washout of SF_6_, but on the entire gas composition, as well as changes in temperature and humidity.

The WBreath algorithm attempts to address this issue with a temperature model that aims to predict the conditions within the flow meter's main compartment for any given time point based on the flow in and out of the sensor and a variety of parameters reflecting ambient conditions (e.g. temperature, time constants, dead space, and physiological volumes, Supporting Information: Table S2).^7^, ^13^

**Figure 1:**
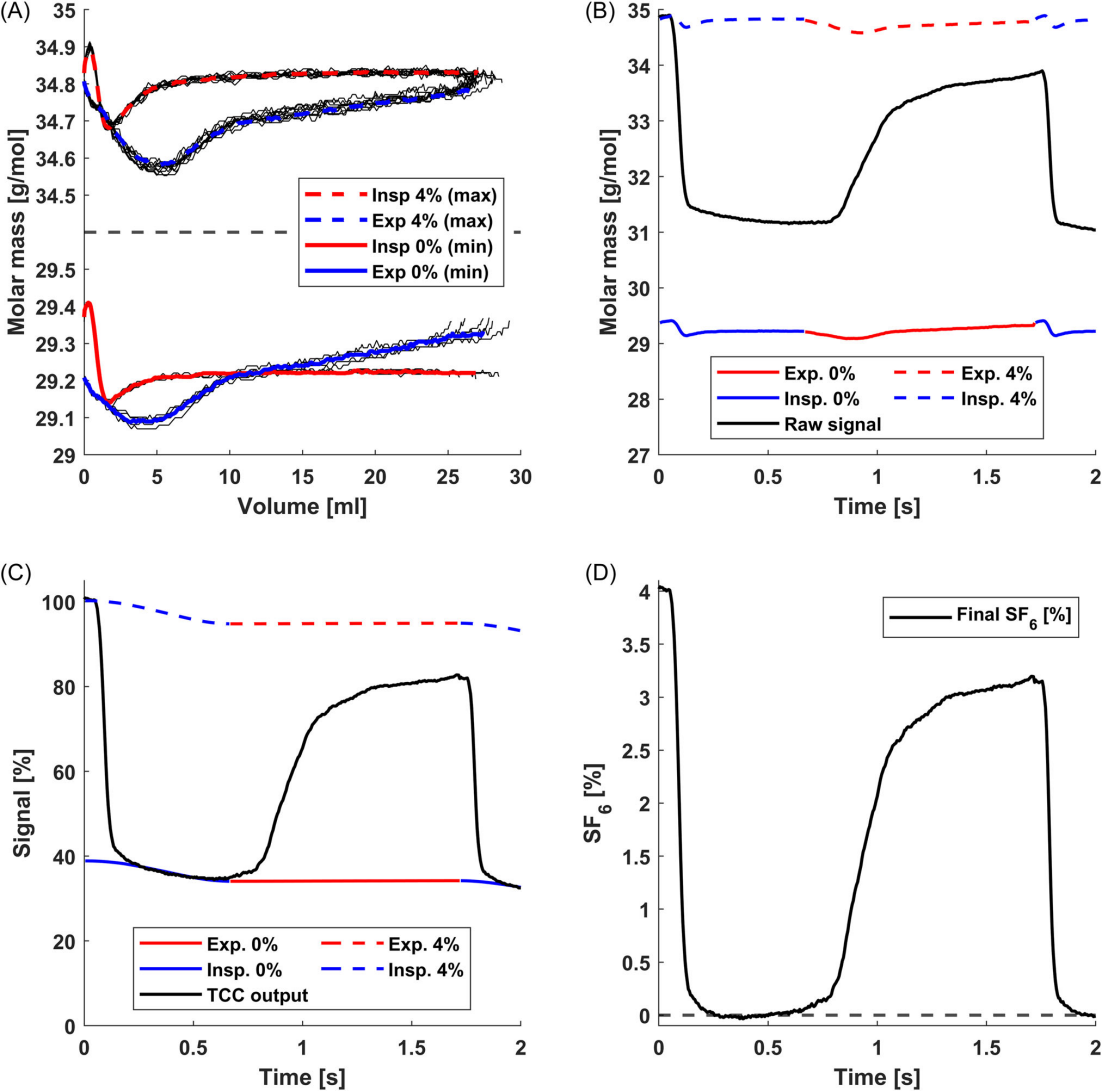
Illustration of the signal processing steps of OASIS. (A) Molar mass (MM) signals during the pre‐phase and end of washin as a function of inspired/expired volume of an example measurement (black lines). From those, a median trace is extracted for each of the four boundary conditions above (expirations/inspirations at minimum/maximum sulfur hexafluoride [SF_6_]). (B–D) Shown is the signal of the first 2 s of the washout phase of an example measurement, showing a complete breathing cycle (inspiration & expiration). (B) Tidal change correction (TCC): Shown is the raw molar mass signal (black), expirogram functions for the end of washin and pre‐phase, and the corresponding inspirogram functions from panel (A). The dashed respirogram functions correspond to the maximum signal (4% SF_6_), the solid lines to the minimum (0% SF_6_). (C) Side chamber correction: Shown is the output of the TCC in panel B) (black). The minimum signal is fit separately to each inspiration as a function of inspired volume, yielding the signal corresponding to 0% SF_6_ during inspirations (solid blue). The discontinuous blue curves are interpolated to yield the curves corresponding to 0% SF_6_ during expirations (solid red). The dashed curves (corresponding to maximum signal) are scaled versions of the solid curves, scaled to ensure that they correctly yield known solutions for the first breath of washin and washout. (D) Final extracted SF_6_ signal (black).

In contrast, OASIS uses the fact that the concentration of SF_6_ is known for certain phases of the measurement, and for those phases it constructs traces of expected molar mass as a function of inspired and expired volume (inspiro‐ and expirograms, Figure 1A). These two conditions are the pre‐phase of the measurement (no SF_6_ present) and the end of washin of SF_6_ (maximum concentration of SF_6_ present, typically 4%). Once constructed, those two boundary conditions can be used as known reference points for the washout breaths. For any given measured time point, given the current inspired or expired volume, the expected molar mass signals for 0% and 4% SF_6_ are known, which allows the calculation of SF_6_ concentration via interpolation (Figure 1A,B).

### 2.6 OASIS: Side chamber correction

The side chambers of the flow meter are a part of the measurement path of the flow meter (Supporting Information: Figure S1), and therefore they contribute to the raw measured speed of sound/molar mass signal. However, those compartments are semi‐accessible to the air, which flows through the main compartment, and a part of that air diffuses into the side chambers with each breath.

The WBreath algorithm attempts to overcome this issue with a diffusion model that aims to predict the conditions within the side chambers for any given time point based on the molar mass signal itself, as well as a variety of parameters governing the diffusion process (Supporting Information: Table S2).^7^

OASIS again uses the signal in those parts of the measurement where the SF_6_ concentration is known.

1. During washout inspirations, the air flowing through the main compartment of the flow meter is known to contain no SF_6_.
2. Additionally, the first breaths of the washin and washout have known concentrations within the main compartment and side chambers.

Under the assumption that the measured molar mass signal is a combination of the signal originating from the main and side compartments, we can calculate the contribution of the side chambers during inspirations. The discontinuous side chamber estimates are interpolated during expirations, to yield an estimate for the side chamber contribution over the whole washout. This estimate can then be used to solve for the main compartment concentration, resulting in a final SF_6_ signal that can be used to calculate MBW outcomes (Figure 1C,D).

### 2.7 Statistics

Primary outcomes of the MBW measurements were the LCI and FRC. Statistics were performed using MATLAB 2021a. To test for normal distribution of data, the Kolmogorov–Smirnov test was used. To test for differences between analysis methods of the same measurement and between longitudinal data points we used the paired *t*‐test. To test for differences between different groups of measurements, we used the unpaired *t*‐test. Significance threshold for statistical testing was defined as *p* = .05. Analysis of variance (ANOVA) was performed using GraphPad Prism 8.0.1 (GraphPad Software), assuming Gaussian distribution of residuals and using the Geisser–Greenhouse correction.

## 3 RESULTS

### 3.1 In vitro validation

**Figure 2:**
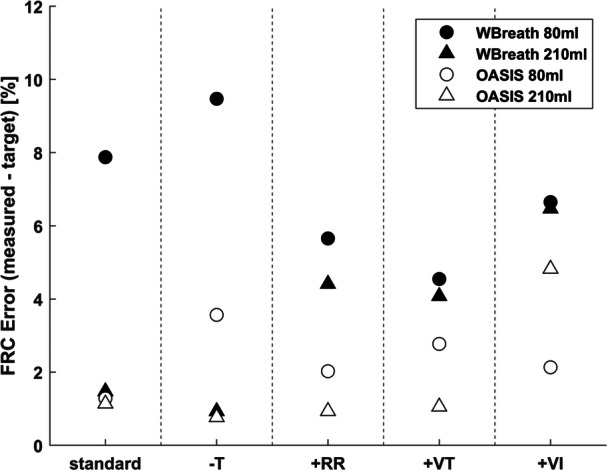
In vitro functional residual capacity (FRC) measurement comparison between WBreath (config II, black markers), and OASIS (empty markers). Shown is the absolute error from the target volume of the in vitro lung model. Data points represent the mean of two triplicates each (initial measurement and repeat on a device calibration). Standard: baseline condition (T = 32.5 ± 1°C, small: FRC = 80 mL, VT = 30 mL, RR = 30/min, large: FRC = 210 mL, VT = 50 mL, RR = 20/min), −T: measurement performed at room temperature, +RR: increased respiratory rate (small: +66%/large: +50%), +VT: increased tidal volume size (small: +66%/large: + 60%), +VI: added ventilation inhomogeneity mesh within lung model.

In the setting of the in vitro lung model, OASIS outperformed WBreath at FRC measurement accuracy in every category examined (Figure 2). The mean (SD) absolute error in FRC measurement decreased from 5.2 (3.2) % to 2.1 (1.6) % (paired *t*‐test: *p* < .001, *n* = 60).

### 3.2 Feasibility and parameter independence

**Figure 3:**
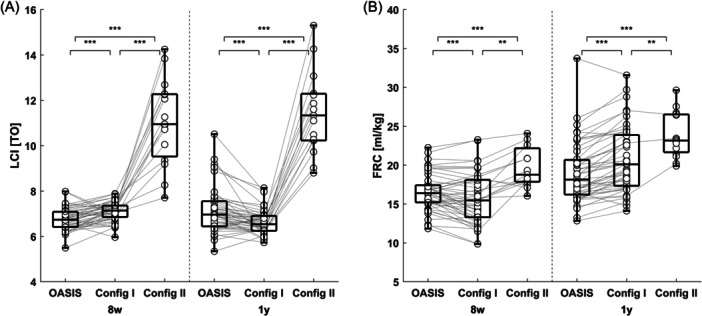
Comparison of infant data using WBreath (config I), WBreath (config II), and OASIS. Shown are the primary outcomes (FRC, LCI) of MBW measurements in *n* = 48 healthy control infants measured at age 8 weeks (8w) and 1 year (1 y) from the Drakenstein cohort (South Africa), analyzed using three different methods. New: Measurements analyzed using OASIS, config I: WBreath with config I settings (Drakenstein cohort settings), config II: WBreath with config II settings (BILD/SCILD cohort settings). Boxes represent median + interquartile ranges. (A) LCI in turnovers (TO) analyzed with different methods, showing the change for each visit between the different methods. (B) FRC in (mL/kg) analyzed with different methods, showing the change for each visit between the different methods. Statistics: Paired *t*‐test: ***p < .001, **p < .01, *p < .05, ns, not significant. BILD, Basel‐Bern Infant Lung Development; FRC, functional residual capacity; LCI, lung clearance index; MBW, multiple‐breath washout; SCILD, Swiss Cystic Fibrosis Infant Lung Development.

When applied to the Drakenstein cohort data, OASIS produced LCI and FRC results that were different from WBreath, both using config I and config II (Figure 3). WBreath also produced different results between config I and config II, showing the importance of temperature and diffusion settings for WBreath. This was the case for data in both 8‐week‐ and 1‐year‐old infants.

Using config II in WBreath, we found valid outcomes in 32% (*n* = 15/48) of individuals, both for 8‐week‐ and 1‐year‐old infants, demonstrating that config II (used by the Switzerland‐based BILD study) has low feasibility in data originating from the Drakenstein cohort *n* = 3 (6%) individuals had valid outcomes for both visits using config II applied to Drakenstein cohort data. Both the config I and OASIS produced valid outcomes for all visits.

### 3.3 Plausibility of results

**Figure 4:**
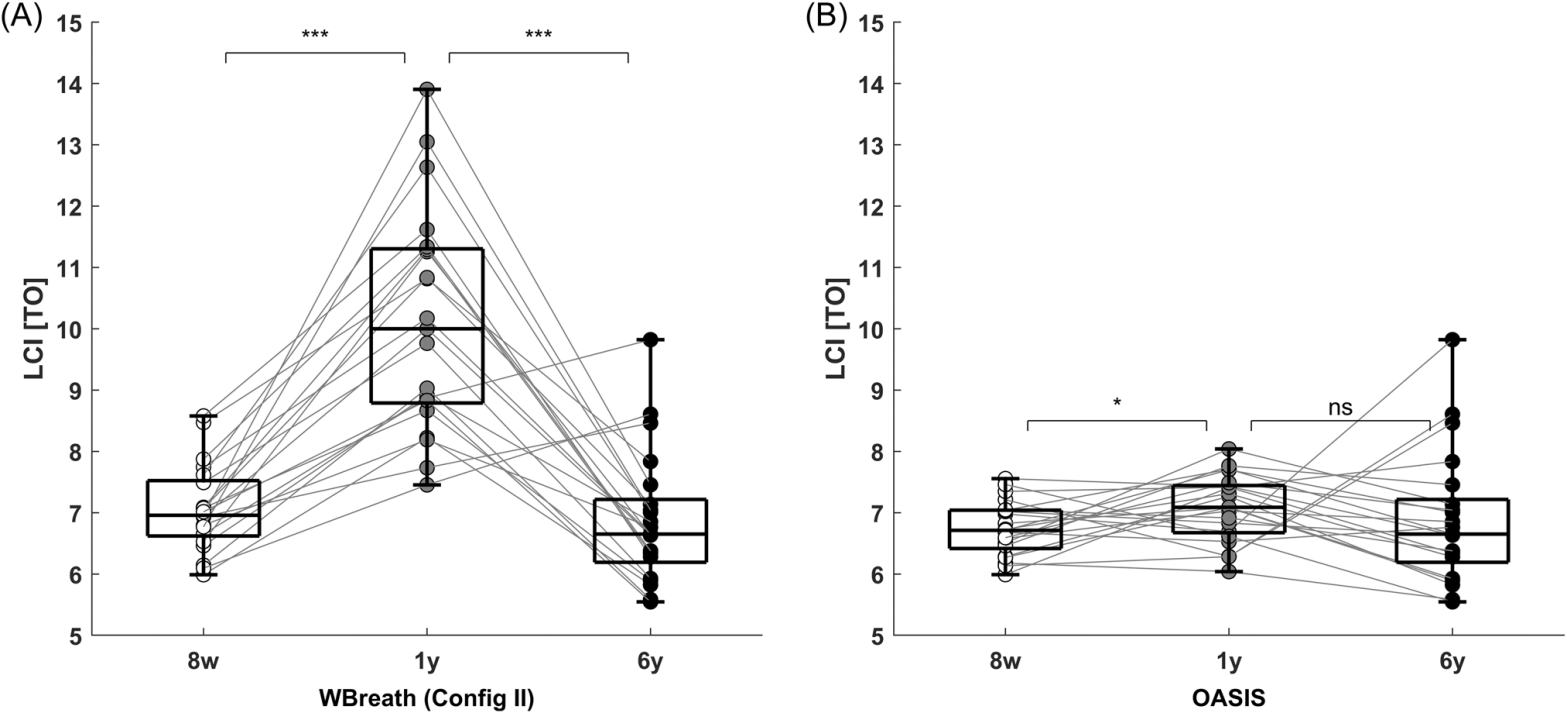
Longitudinal change in lung clearance index in children with cystic fibrosis. Shown is lung clearance index (LCI) in turnovers (TO) for *n* = 21 children from the Basel‐Bern Infant Lung Development (BILD) cohort (Bern) measured at 8 weeks (8w, infant sulfur hexafluoride [SF_6_] multiple‐breath washout [MBW], either WBreath or OASIS), 1 year (1 y, infant SF_6_ MBW, either WBreath or OASIS), and at 6 years (6 y, N_2_ MBW, Exhalyzer D, Spiroware 3.3.1). Boxes show median + interquartile ranges. (A) Longitudinal analysis using WBreath for infant MBW measurements. (B) Longitudinal analysis using OASIS for infant MBW measurements. Statistics: Paired *t*‐test, ***p < .001, *p < .05, ns, not significant.

In SCILD cohort children (Bern, CF), WBreath (config II) showed a 42% increase in mean (SD) LCI going from 7.1 (0.7) Turnovers (TO) at 8 weeks to 10.1 (1.8) TO at 1‐year‐old (paired *t*‐test: *p* < .001), which then decreased again by 32% at the 6 year follow‐up, to 6.9 (1.1) TO (paired *t*‐test: *p* < .001, Figure 4A).

OASIS showed a more plausible steady progression of mean (SD) LCI values over time, going from 6.7 (0.4) TO at 8 weeks to 7.1 (0.5) TO at 1 year (Paired t‐test: *p* = 0.03), then to 6.9 (1.1) TO at 6 years (paired *t*‐test: *p* = 0.46, Figure 4B). One‐way repeated measures analysis of variance (ANOVA) showed significant differences between longitudinal groups analyzed using WBreath (*p* > .001), but not when analyzed with OASIS (*p* = .33).

**Figure 5:**
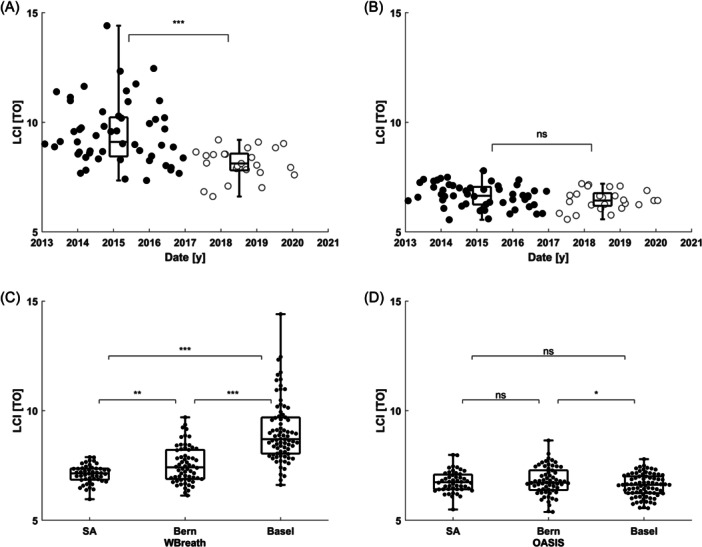
Multiple‐breath washout (MBW) outcomes in healthy control 6‐week‐old infants over time and across centers. (A and B) Shown is lung clearance index (LCI) in turnovers (TO) for *n* = 78 children from the Basel‐Bern Infant Lung Development (BILD) cohort (Basel) measured at 6 weeks, as a function of test date. Black dots indicate measurements before January 2017 (*n* = 53), empty circles measurements after January 2017. (A) Analysis using WBreath. (B) Analysis using OASIS. (C and D) Shown is LCI in TO for infants in South Africa (*n* = 48, Drakenstein cohort, 8 weeks old), Bern (*n* = 62, BILD cohort, 6 weeks old) and Basel (*n* = 78, BILD cohort, 6 weeks old). (C) Center comparison using WBreath (SA: config I, Bern: config II, Basel: config II). (D) Center comparison using OASIS. Boxes show median + interquartile ranges of the two sub‐groups. Statistics: Unpaired *t*‐test ***p < .001, **p < .01, *p < .05, ns, not significant.

In BILD cohort data measured in Basel, WBreath (config II) showed an unexplained decrease in mean (SD) LCI values when comparing data from before 2017 and 2017 and after (before: 9.6 (1.5) TO, after: 8.1 (0.7) TO, paired *t*‐test: *p* < .001). OASIS showed no change over time (before: 6.7 (0.5) TO, after: 6.5 (0.5) TO, paired *t*‐test: *p* = .13, Figure 5A,B).

LCI values in healthy children from three different centers measured in the infant age range (Basel and Basel: around 6 weeks, South Africa: around 8 weeks) showed large differences when analyzed with WBreath (difference in mean LCI, *p*‐value unpaired *t*‐test: Bern‐South Africa: 0.44 TO, *p* = .001; Basel‐Bern: 1.5 TO, *p* < .001; Basel‐South Africa: 1.9 TO, *p* < .001; Figure 5C and Supporting Information: Table S3). These same differences were greatly reduced when the same measurements were analyzed using OASIS (difference in mean LCI, *p*‐value unpaired *t*‐test: Bern‐South Africa: 0.04 TO, *p* = .71; Basel‐Bern: −0.21 TO, *p* = .04; Basel‐South Africa: −0.17 TO, *p* = .08; Figure 5D and Supporting Information: Table S3).

## 4 DISCUSSION

### 4.1 Summary

We developed an improved method for speed‐of‐sound‐based infant SF_6_ MBW measurements. This improved algorithm allows a single method to analyze measurements from different age groups and centers without the need to adjust and validate model input parameters. This represents a meaningful advance in being able to obtain the best possible results from a legacy system of lung function measurement.

### 4.2 Applicability of the algorithm

While OASIS is no longer reliant on a large number of input parameters to perform its corrections, it uses assumptions which are specifically tailored to infant SF_6_ MBW. To perform the tidal change correction, it makes use of the fact that breathing in sleeping infants is more regular compared to breathing in awake, older children.

The tidal change correction also relies on the assumption that O_2_/CO_2_ exchange remains steady throughout a measurement. Due to the presence of only a single sensor to measure the gas composition, there is not enough information present in molar mass‐based infant SF_6_ measurements to disentangle rising and falling concentrations of SF_6_ and CO_2_.

In addition, the algorithm relies on the assumption that the washin of SF_6_ is complete and the concentration of the tracer gas is at 4%. Visual and/or numerical quality control would therefore need to be performed to ensure these assumptions are valid. Variance in the concentration of the tracer gas supply would lead to errors in FRC estimation proportional to the variance, while incomplete washin would lead to overestimated SF_6_ concentrations.

The required signals of the algorithm (flow and molar mass signals) are also present in the more modern Exhalyzer D system for infant MBW, so it could theoretically be applied to raw data originating from that system. The utility of doing so may however be limited, as the Exhalyzer D device has additional sensors which overcome limitations of previous set‐ups, and has recently undergone software improvements^22^ and validation^23^ that promise to make it suitable for use moving forward. The algorithm also relies on the presence of both a washin and washout phase, and cannot be generalized to analyze washout‐only N_2_MBW measurements.

### 4.3 Discussion of results

We showed that this difficulty of finding the right parameters for WBreath is highly relevant in vivo, by analyzing data originating from the Drakenstein cohort with both the settings typically used in South Africa and Switzerland, and showing that results differed greatly between the two settings. This non‐compatibility of the same method with the environmental conditions of the respective centers had previously made comparison of data between centers very difficult.

OASIS proved to be adaptable to the different environmental conditions, but also produced significantly different results from both of the WBreath methods. Importantly, within a single age group in a single center, there are still differences in breathing pattern and environmental influences which may not be sufficiently taken into account by a single set of WBreath model input parameters. OASIS improves on this by constructing its corrections from each individual measurement.

We also showed that OASIS produces more plausible LCI values in the longitudinal analysis of data from infants with CF. While WBreath produced results that implied that ventilation inhomogeneity peaked far above the healthy range at 1 year of age, and then normalized again when measured at 6 years, OASIS showed that ventilation inhomogeneity was more steady and comparable leading up to the age of 6 years.

OASIS also showed more stability in its results over time, in data from healthy children from Basel. WBreath seemed to show that LCI values in that data showed a significant drop which did not correlate with any known change in environment or measurement setup. OASIS showed no such influence, indicating that it is more stable when faced with minor changes in measurement setup than WBreath.

Finally, OASIS produced results which were much more similar between centers for similar age groups of healthy children between centers, while also reducing abnormally high LCI values down to a healthier range.

### 4.4 Comparison with other studies

Previous work largely aimed to improve the analysis of molar mass‐based infant SF_6_ MBW measurements by aiming to better characterize WBreath or modify parameters to produce improved results.^6^, ^7^, ^15^

In contrast, this manuscript presents a fundamentally new approach to the signal processing of the molar mass signal. It provides a fully transparent algorithm for researchers to apply to their data, and greatly reduces sources of error by replacing the need for a large set of input parameters with information inferred from the measurement itself.

### 4.5 Strengths and limitations

We were able to validate the new algorithm both in vitro and in vivo, in longitudinal data across the infant age range, as well as in healthy children and children with CF.

While the good performance of OASIS at measuring FRC in the in vitro setting is encouraging, the lung model did not include a simulation of O_2_/CO_2_ exchange. The performance of WBreath in that setting may also have been suboptimal due to the difficulty of adjusting the model parameter settings to the altered setup.

Further, the new algorithm relies on assumptions which may introduce error into the measurement. The applicability of some of these assumptions can be verified through quality control (e.g., for sufficient washin of tracer gas and a steady breathing pattern), but for others the necessary information is missing (steady O_2_/CO_2_ exchange throughout the measurement).

However, while OASIS produced overall more plausible results than WBreath in vivo, there is no true gold standard for LCI in vivo we could validate it with. Future studies could compare LCI measured with OASIS to LCI measured with respiratory mass spectrometry, or with more modern infant MBW systems such as the Exhalyzer D.

### 4.6 Meaning and implication/outlook

The broad applicability of a single algorithm to any kind of molar mass‐based infant SF_6_ MBW, which is fully transparent and openly available, constitutes a breakthrough for a method which has long been held back by technical limitations in analysis software. Different studies across the globe have measured thousands of infant SF_6_ MBW measurements,^10^, ^19^, ^20^ and this work serves to enable the researchers who use this data to obtain reliable results from those measurements.

## 5 CONCLUSION

OASIS achieved improved accuracy in FRC measurement in vitro, as well as improved comparability between age groups and research centers in vivo. Thus, this algorithm represents a plausible solution to improving the analysis of the infant MBW data sets collected using ultrasonic flow meters.

## AUTHOR CONTRIBUTIONS

**Florian Wyler**: Conceptualization; methodology; software; writing—original draft; visualization; formal analysis; data curation; investigation. **Thuvarakha Manogaran**: Resources; writing—review & editing; investigation; methodology; validation. **Nathalie Monney**: Validation; investigation; formal analysis; methodology; data curation. **Yasmin Salem**: Investigation; writing—review & editing; data curation. **Ruth Steinberg**: Writing—review & editing; investigation; data curation. **Anne‐Christianne Kentgens**: Writing—review & editing; investigation; data curation. **Carvern Jacobs**: Investigation; writing—review & editing; data curation. **Shaakira Chaya**: Writing—review & editing; investigation; data curation. **Carla Rebeca da Silva Sena**: Writing—review & editing; investigation; data curation. **Noëmi Künstle**: Writing—review & editing; investigation; data curation. **Olga Gorlanova**: Writing—review & editing; investigation; data curation. **Sophie Yammine**: Supervision; writing—review & editing; data curation. **Urs Frey**: Project administration; writing—review & editing; funding acquisition; supervision. **Marc‐Alexander Oestreich**: Investigation; methodology; data curation; writing—review & editing. **Philipp Latzin**: Conceptualization; writing—review & editing; methodology; supervision; project administration; funding acquisition; resources.

## CONFLICT OF INTEREST STATEMENT

Urs Frey and Philipp Latzin received grants from the Swiss National Science Foundation (182719, 204717). Anne‐Christianne Kentgens received support from the Excellence Scholarship by the Swiss Confederation. Marc‐Alexander Oestreich is deputy speaker of the working group on pediatric lung function of the German Society for Pediatric Respiratory Medicine. Philipp Latzin receives fees from Vertex, OM Pharma, Vifor, Polyphor, Santhera (DMC), Allecra, and Sanofi Aventis. Florian Wyler, Marc‐Alexander Oestreich, and Philipp Latzin are in regular contact with manufacturers of MBW devices. Florian Wyler was temporarily employed by ndd Medizintechnik AG (Zurich, Switzerland) in August 2022 for an unrelated project. Ecomedics AG (Duernten, Switzerland) provided assistance in the form of a calibrated syringe pump. The Drakenstein child health study was supported by the Wellcome Trust (#098479/z/12/z), Bill and Melinda Gates Foundation (OPP1017641), and Thrasher Foundation (#9207).

## ETHICS STATEMENT

Study protocols were approved by the ethics committees of the Canton of Bern (2019‐01072, 2017‐02139), Northwest and Central Switzerland (2022–00336) as well as the Western Cape Provincial Health Research Committee and University of Cape Town Human Research Ethics Committee (401/2009).

## Supporting information

Supporting information.

Supporting information.

## Data Availability

The data that support the findings of this study are available on request from the corresponding author. The data are not publicly available due to privacy or ethical restrictions.
