# Supplementary material for: Optimized algorithm for speed‐of‐sound‐based infant sulfur hexafluoride multiple‐breath washout measurements
Source: Pediatr Pulmonol. 2024 Jul 18;59(12):3240–9. doi: 10.1002/ppul.27180 (PMC11601022; doi:10.1002/ppul.27180)
Supplement: Supplementary file 1 — Supporting information. [file PPUL-59-3240-s002.docx]

**Supplemental Table S1: Measurement conditions for *in vitro* lung model validation of FRC measurement.** Abbreviations: FRC: functional residual capacity, T: temperature of the water bath surrounding the lung model, either heated to 32.5±1 °C (body), or unheated (room); RR: respiratory rate; VT: tidal volume; VI: ventilation inhomogeneity insert present.

**Supplemental Table S2: Overview over configurations used as inputs to WBreath**. Config I has been used in South Africa to analyze measurements of the Drakenstein cohort, while config II is the standard in Switzerland for analyzing BILD and SCILD cohort measurements. Differences in parameters for the same age group represent historical adaptations of the methodology made to adapt to local climate conditions and/or to ensure feasibility of measurements. Infant: Settings used for 5-8-week-old infants, Toddler: settings used for 1-year-old infants. Bold entries represent settings with a discrepancy or adaptation between the centers. Abbreviations: Var: adjusted individually. Meas: measured by the setup. EIMM: End-inspiratory molar mass difference used as reference for 4% SF_6_ molar mass difference. Cutting: Additional processing step performed, where end-of-test breath is determined, measurement is cut at 10 breaths after end-of-test, and analysis is re-performed.


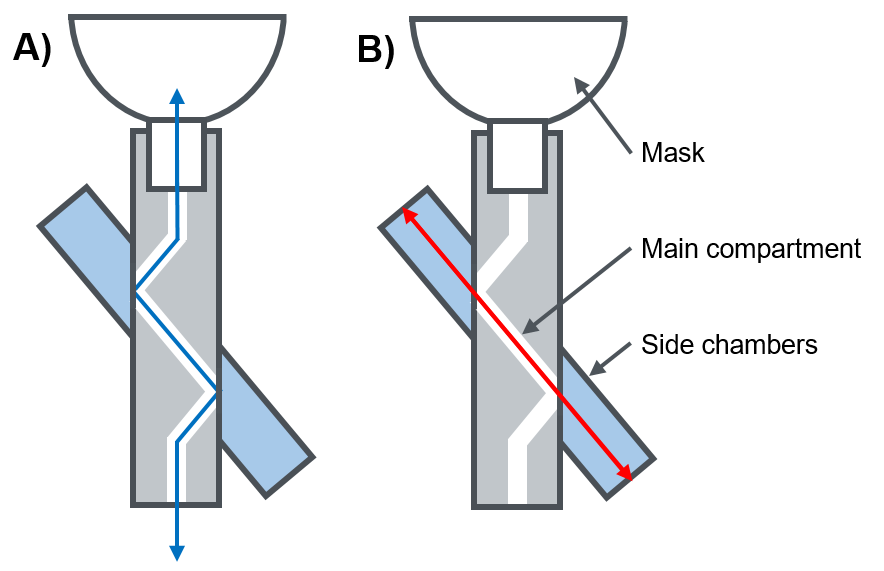


**Supplemental Figure S1: Diagram of the ultrasonic flow meter used to collect flow and molar mass raw data**. A) Inspired/Expired air travels along the main path marked with blue arrows. B) Ultrasonic pulses are sent back and forth along the path marked with red arrows. Side chambers are marked in blue. The current flow and speed of sound of the medium within the travel path (red) can be inferred from the difference in travel times of the ultrasonic pulses, as well as their total travel time. From the speed of sound, a raw molar mass signal is inferred according to Equation 1.

**Supplemental Table S3: MBW outcome comparison between centers in 6-week-old healthy infants.** Shown is mean (SD) lung clearance index (LCI) in turnovers [TO] and FRC in [ml/kg] for infants in South Africa (n = 48, Drakenstein cohort, 8 weeks), Bern (n = 62, BILD cohort, 6 weeks) and Basel (n=78, BILD cohort, 5 weeks). WBreath (SA: config I, Bern: config II, Basel: config II)
